# “Brake” and “accelerator”: revisiting tumor cell direct responses and the paradox of aggression in anti-VEGF therapy

**DOI:** 10.3389/fimmu.2025.1718894

**Published:** 2026-01-16

**Authors:** Pei Wei, Jiaqi Li, Xueyan Cheng, Yanxin Lu, Qiang Xia, Zhiyong Wang

**Affiliations:** Department of Immunology, Zunyi Medical University, Zhuhai, China

**Keywords:** anti-VEGF therapy, direct signal remodeling, paradox of aggression, therapeutic resistance, vascular endothelial growth factor

## Abstract

Targeted anti-angiogenic therapies against vascular endothelial growth factor (VEGF) are standard treatments for various advanced cancers. However, their clinical benefits are often limited by acquired resistance, with some patients even exhibiting paradoxical tumor aggressiveness and accelerated metastasis. Traditional views primarily attribute this to hypoxia-driven mechanisms within the tumor microenvironment. Based on an analysis of receptor tyrosine kinase (RTK) interactions and pathway rewiring within tumor cells, this paper proposes an integrated “brake-accelerator” model. We posit that in the presence of VEGF, VEGFR2–Mesenchymal-Epithelial Transition factor (MET) heterocomplexes and neuropilin (NRP) co-receptors restrict bypass pathway activity. Upon VEGF blockade, this “brake” is released, and bypass “accelerators” are passively or actively amplified, collectively driving invasive transformation and immune evasion. We identify “direct signal remodeling” in tumor cells as a potentially underappreciated contributor. This process acts as a potential upstream integration node that, alongside treatment-induced hypoxic stress and immune–stromal microenvironment remodeling, constitutes a “tripartite stress” framework. Through Darwinian clonal selection and induced phenotypic plasticity, this framework drives the evolution of tumors towards a more aggressive phenotype. This paper systematically reviews the molecular mechanisms supporting this model, including the regulatory role of VEGFR2-MET complexes, the signal hub function of NRP, and the networked characteristics of escape pathways. Finally, we discuss the implications of this conceptual model for future clinical practice, including the development of dynamic biomarkers based on intrinsic tumor cell characteristics and the design of more precise combination and adaptive treatment strategies to overcome resistance to anti-VEGF therapy and improve patient prognosis.

## 1 Introduction

Inhibition of tumor angiogenesis has been one of the most significant advances in oncology over the past two decades (1–3). Anti-angiogenic agents, exemplified by the humanized anti-VEGFA monoclonal antibody bevacizumab, have become a cornerstone of treatment for various solid tumors, including colorectal cancer (CRC) (4, 5), non-small cell lung cancer (NSCLC) (6, 7), and glioblastoma (GBM) (8, 9), significantly extending patients’ progression-free survival (PFS). However, the long-term efficacy of this strategy has not met initial expectations. The vast majority of patients eventually develop acquired resistance, and the improvement in overall survival (OS) has been modest (10, 11).

More concerning is the “paradox of aggression” observed in preclinical models and some retrospective clinical studies. In these cases, although tumors were initially controlled radiographically after anti-VEGF therapy, the recurrent disease exhibited enhanced local invasion and distant metastatic potential (12, 13). For instance, in patients with recurrent GBM, the non-enhancing, diffuse infiltrative recurrence pattern observed after bevacizumab treatment portends a worse prognosis (14–16). This phenomenon suggests that while anti-VEGF therapy inhibits tumor angiogenesis, it may inadvertently activate molecular programs that promote malignant progression.

To explain the resistance and paradox of aggression, several non-mutually exclusive models have been proposed. Model 1: The classic hypoxia-driven model. This model posits that anti-angiogenic therapy exacerbates tumor hypoxia, thereby upregulating hypoxia-inducible factors (HIFs). This in turn activates alternative pro-angiogenic factors [e.g., fibroblast growth factors (FGF), platelet-derived growth factors (PDGF)] and pro-invasion/metastasis pathways [e.g., mesenchymal-epithelial transition factor (MET), AXL receptor tyrosine kinase (AXL)] to compensate for the loss of VEGF signaling (17–19). Model 2: The metabolic/autophagy reprogramming model. This model focuses on metabolic shifts in tumor cells—such as switching to oxidative phosphorylation or enhancing autophagy—to adapt to limited nutrient and oxygen supply (19–21). Model 3: The immune microenvironment regulation model. This model posits that while VEGF blockade might improve T cell infiltration, it could also lead to the enrichment of pro-angiogenic and immunosuppressive myeloid-derived suppressor cells (MDSCs), thereby fostering resistance (22–24). Model 4: The direct signaling model. This model acknowledges that many tumor cells express VEGF receptors (VEGFRs), and thus anti-VEGF drugs may directly influence tumor cell behavior (25–27).

While these models elucidate partial truths, they predominantly regard tumor cells as passive adapters, failing to fully integrate the direct and active regulatory roles of anti-VEGF drugs on intrinsic tumor cell signaling. Building upon this, this paper proposes a more comprehensive “direct signal remodeling” framework. We argue that the direct effect of anti-VEGF drugs on tumor cells (i.e., Model 4) should not be viewed as merely one of several parallel mechanisms. Instead, we propose that this drug-induced signal network remodeling could be an early organizing node that helps orchestrate other adaptive changes, including treatment-induced hypoxic stress and immune microenvironment remodeling. The precise temporal hierarchy between these processes, however, remains to be fully elucidated, and we therefore present this as a working model rather than a proven sequence. This provides a more causally-linked, integrated framework.

We contend that the effects of anti-VEGF therapy extend far beyond indirect “angiocentricity”; in addition to their vascular effects, these agents can also profoundly and directly influence VEGFR-expressing tumor cells. This influence is dual-faceted: on one hand, it inhibits VEGF-dependent survival signals. On the other hand, and equally importantly, it may release a constitutive brake that VEGF signaling imposes on key pro-invasive pathways. A typical example is the disruption of the VEGFR2-MET complex, which liberates MET’s pro-invasive activity (28). This therapy-induced direct signal remodeling, coupled with the ensuing hypoxic stress and immune–stromal microenvironment changes, collectively forms a “tripartite stress” framework. Through Darwinian selection and induced phenotypic plasticity, this framework ultimately drives tumor evolution toward a more aggressive phenotype. Understanding this process is key to overcoming current therapeutic dilemmas.

## 2 Core principles of direct VEGF signaling

In this section, we outline four tumor cell–centric principles that explain why anti−VEGF therapy can paradoxically promote invasive escape. We progress from (1) tumor cells as direct and semi−enclosed VEGF targets, to (2) a prototypical brake–accelerator module (VEGFR2–MET), (3) networked escape via NRP−centered hubs and non−canonical VEGF signaling, and (4) evolutionary enforcement through clonal selection and induced plasticity, which together set the stage for the integrative framework in Section 3.

### 2.1 Tumor cells as direct and semi-enclosed VEGF targets

Traditionally, anti-VEGF therapy has been understood as targeting endothelial cells. However, abundant evidence indicates that various tumor cell types—including GBM, CRC, breast cancer, and renal cancer—themselves express functionally relevant levels of VEGF receptor family members such as VEGFR1, VEGFR2, and the co-receptors neuropilins (NRPs) (29–32). These receptors are not “silent” but constitute functionally active signal transduction systems. On one hand, tumor cells secrete VEGF, forming autocrine or paracrine loops that directly promote their own proliferation, survival, and migration (33, 34). More importantly, several studies have uncovered an intracrine VEGF signaling loop, in which newly synthesized VEGF can directly bind and activate VEGFRs on organelles like the endoplasmic reticulum or Golgi apparatus, without prior secretion, thereby maintaining cell survival and motility in certain tumor contexts (35–38). Collectively, these findings suggest that autocrine/intracrine VEGF signaling is observed across multiple solid tumor types, although its prevalence and functional importance appear to be context-dependent and require further clinical validation.

This “semi-enclosed” signaling system, particularly the intracrine loop, may provide a mechanism contributing to resistance against antibody drugs like bevacizumab, which only neutralize extracellular VEGF. More critically, its importance extends beyond explaining partial resistance. This suggests a core biological context wherein the VEGFR system in tumor cells can function as a continuously active, intrinsic regulatory hub. This persistent endogenous signaling implies that even when extracellular VEGF is blocked, intracellular signaling may remain active, at least transiently, potentially maintaining the suppression of certain bypass pathways. Because tumor cells are direct and complex targets of VEGF signaling, we must understand how their internal signaling network is remodeled upon therapeutic blockade. This leads directly to our core mechanism—the “brake-accelerator” effect.

### 2.2 A prototypical brake–accelerator module: the VEGFR2–MET complex

**Figure 1:**
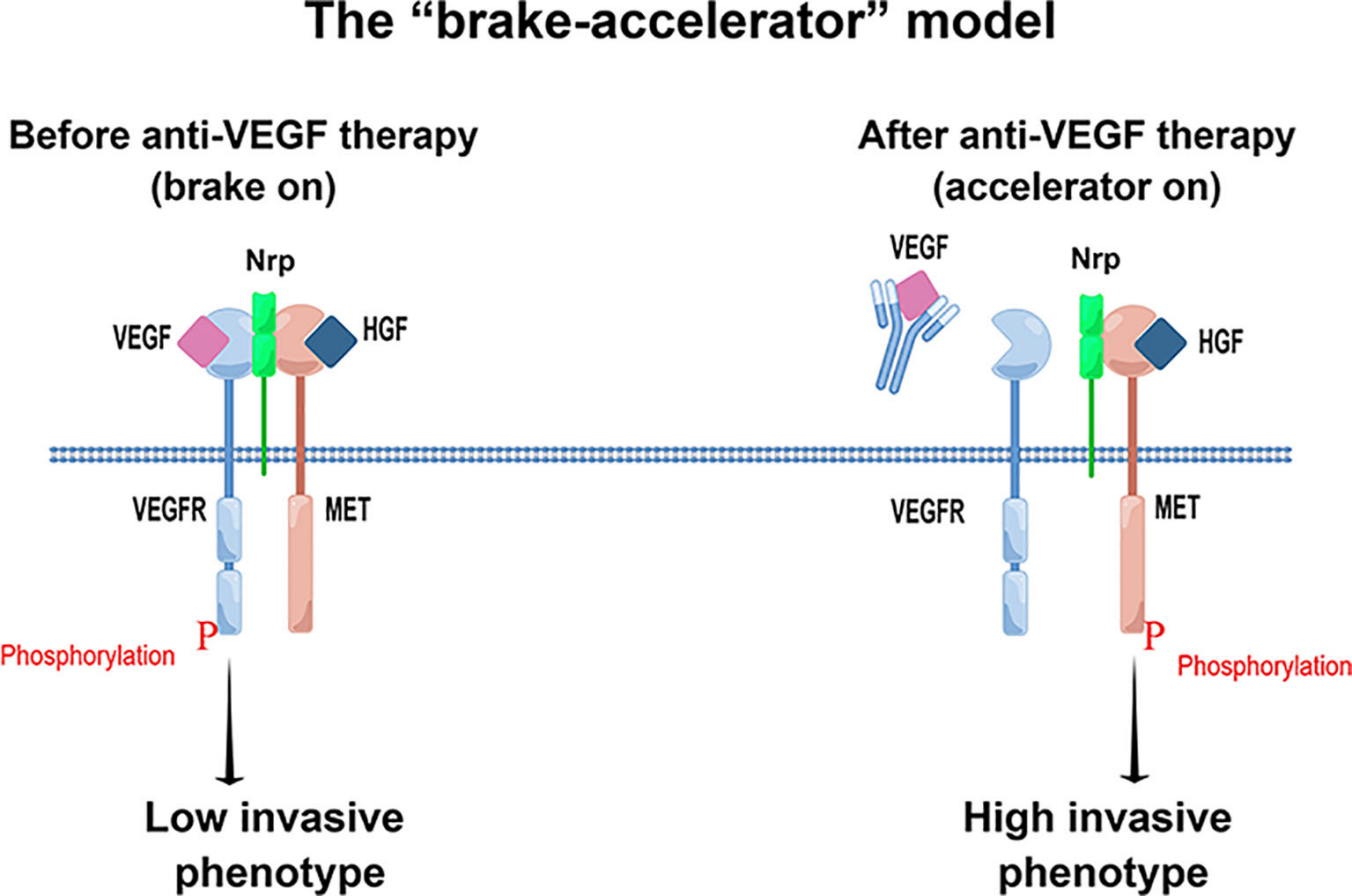
The “brake-accelerator” model of direct signal remodeling involving Nrp in response to anti-VEGF therapy. This model illustrates how anti-VEGF therapy can paradoxically promote tumor aggressiveness by directly altering the interplay between RTKs and their co-receptors on the tumor cell surface. (Left) Before anti-VEGF therapy (brake on): In the presence of VEGF, its binding to VEGFR promotes the recruitment of Nrp, forming a VEGFR-Nrp-MET heterocomplex. This ternary complex acts as a “brake,” constraining MET-driven pro-invasive signaling pathways and thus maintaining a low invasive phenotype. (Right) After anti-VEGF therapy (accelerator on): Anti-VEGF agents prevent VEGF from binding to its receptor, leading to the dissociation of the ternary complex. However, Nrp remains associated with MET, acting as a signaling hub to stabilize MET or facilitate HGF binding. This distinct reconfiguration “releases the brake” on MET, which then becomes phosphorylated and activated, functioning as an “accelerator” to potently drive downstream signaling and result in a high invasive phenotype.

Against this backdrop of direct and semi-enclosed VEGF signaling in tumor cells, the effect of VEGF signaling on tumor cells is bidirectional and context-dependent. Beyond its classic role as a pro-survival “accelerator,” it can also function as a “brake” inhibiting invasion. A central feature of this proposed mechanism appears to be the dynamic interaction between receptor tyrosine kinases (RTKs). A landmark study in GBM found that VEGF promotes the formation of a heterocomplex between VEGFR2 and MET (the receptor for hepatocyte growth factor, HGF). Mechanistically, ligand-activated VEGFR2 recruits protein tyrosine phosphatases (such as PTP1B) to the complex, which dephosphorylates MET and suppresses its kinase activity (28). This suppression effectively halts downstream pro-invasive and epithelial-mesenchymal transition (EMT) signaling (28). Consequently, when anti-VEGF drugs block this pathway, the heterocomplex dissociates. This “releases the brake,” liberating suppressed MET signaling and powerfully driving tumor invasion (**Figure 1**). It should be noted that while research demonstrates this “suppression-release” mechanism in various models (28, 39), its universality and effect intensity across different cancer types are not yet confirmed with equivalent evidence. At present, the VEGFR2–MET heterocomplex is therefore best viewed as a prototypical “brake module” rather than the only form of VEGF-mediated restraint. Emerging data suggest that VEGF/VEGFR signaling can functionally interact with other pro-invasive RTKs—including AXL, fibroblast growth factor receptors (FGFRs), and the epidermal growth factor receptor (EGFR)—through diverse mechanisms such as reciprocal transcriptional regulation, shared adaptor proteins, and co-localization within membrane microdomains (40–43). Whether any of these interactions involve analogous cis-inhibitory complexes remains to be determined and represents an important area for future investigation. In the following sections, we use the VEGFR2–MET module as a mechanistically well-characterized example to illustrate how direct VEGF blockade can shift the balance from “brake” to “accelerator”, while acknowledging that additional, parallel restraints may exist within the broader RTK network.

### 2.3 Networked escape and NRP-centered signal hubs

Tumor cells exhibit high signaling plasticity in response to VEGF pathway blockade. The escape process may be better conceptualized not as a linear compensation, but rather as a multi-node, networked remodeling. The activation of the MET pathway is the most studied example but represents only a fraction of the network (44, 45). This network encompasses at least three levels: 1) “Brake-release” mechanisms, such as the aforementioned MET signal liberation (28, 39, 44–49); 2) “Bypass activation,” involving the transcriptional upregulation of other RTKs like FGFR, EGFR, platelet-derived growth factor receptor (PDGFR) (18, 50–53); and 3) “Hub amplification,” which upregulates key signal integrators.

Within this escape network, NRP-1 and NRP-2 are critical signaling and network amplification hubs. As key VEGF co-receptors, they regulate stemness, migration, and invasion, while also integrating anti-VEGF pressure with other signals (54–56). Specifically, NRP1 is often associated with angiogenesis, stemness, and transforming growth factor-β (TGF-β)-mediated fibrosis/immunosuppression (57, 58), whereas NRP2 is more prominent in metastasis and lymphangiogenesis (59, 60). Both can crosstalk with the HGF/MET, Ephrin-Eph, and TGF-β networks and promote EMT and metabolic plasticity by linking to the phosphatidylinositol 3-kinase (PI3K)/AKT and mitogen-activated protein kinase (MAPK) axes via integrins/focal adhesion kinase (FAK). Although early NRP inhibitors showed activity, safety and druggability remain challenges (61, 62). Accumulating preclinical work and clinical cohort analyses in several solid tumors support a prognostic—and in some settings potentially predictive—role for NRP1/2 expression, but robust, prospective validation in large trials is still lacking. For instance, a systematic review and meta-analysis confirmed that high NRP1 expression was associated with worse overall survival in both hepatocellular and colorectal cancer cohorts (63). Furthermore, a correlative analysis of the landmark IMvigor210 trial in muscle-invasive bladder cancer revealed that high NRP1 expression not only predicted poor survival but was also associated with resistance to PD-L1 blockade, linking it to an immunosuppressive tumor microenvironment (64). Therefore, we propose that NRPs should be prioritized as key biomarkers and co-inhibitory targets for further validation in sequential combinations with MET/AXL or TGF-β pathway inhibitors.

Beyond canonical VEGFR2-dependent signaling, non-classical VEGF pathways further expand this networked escape. VEGFR1, often described as a regulatory or “decoy” receptor, can nonetheless transduce pro-migratory and pro-invasive signals in certain metastatic models and may modulate the availability of VEGF for VEGFR2 and NRP complexes (65–68). In parallel, NRPs can mediate VEGF-dependent but VEGFR2-independent signaling, and can also engage ligands such as TGF-β, HGF, and class 3 semaphorins to drive tumor growth, DNA damage repair, and therapy resistance even when VEGFR2 is pharmacologically inhibited (69–71). These non-canonical routes provide additional conduits for sustaining invasive and adaptive signaling under VEGF/VEGFR2 blockade and should be considered integral components of the “network remodeling” framework.

### 2.4 Evolutionary enforcement: clonal selection and induced plasticity

Anti-VEGF therapy acts as a powerful selector, driving the overall malignant evolution of tumors through two parallel and non-mutually exclusive mechanisms. The first mechanism is classic Darwinian clonal selection. In the early stages of treatment, tumor cell clones highly dependent on VEGF autocrine signaling are effectively eliminated, leading to tumor volume reduction. However, pre-existing minority cell clones within the tumor, independent of VEGF signaling or already having activated aggressive pathways such as MET/NRP, gain a significant survival advantage under this selective pressure and eventually proliferate and thrive, becoming the predominant component of recurrent tumors (28, 50, 72). The second mechanism is induced phenotypic plasticity. Therapeutic pressure itself can serve as a potent environmental signal, actively inducing molecular and phenotypic reprogramming in tumor cells. This includes: 1) EMT, which confers motility and invasive capabilities to cells (73, 74); 2) Acquisition of stemness, enabling cells to possess stronger self-renewal and drug resistance (56, 75); 3) Metabolic plasticity, whereby tumor cells adjust their energy metabolism patterns to adapt to resource-scarce environments (19–21). For instance, in GBM models, treatment with the anti-angiogenic drug sunitinib has been found to induce tumor cells to accumulate stem cell markers and exhibit a mesenchymal phenotype (75). These two mechanisms operate in parallel, jointly shaping tumors that are more aggressive and resistant after treatment, and they provide the evolutionary foundation for the “tripartite stress” framework integrating direct signal remodeling, hypoxia, and immune–stromal reprogramming discussed in the following section.

## 3 Integration and new framework: a “tripartite stress” framework

To more comprehensively understand the malignant evolution of tumors after anti−VEGF therapy, we propose a “tripartite stress model.” This framework explicitly integrates the tumor cell–intrinsic mechanisms detailed in Section 2 (direct signal remodeling and evolutionary enforcement) with the extrinsic pressures of the tumor microenvironment. We posit that three mutually coupled selective stresses act on VEGFR−expressing tumor cells and their microenvironment, and—via clonal selection and phenotypic plasticity—drive tumor evolution toward invasive, therapy−resistant states. Stress 1: Exogenous hypoxic stress. This is the most direct consequence of anti-angiogenic treatment. Vascular regression intensifies intratumoral hypoxia and activates HIF-1α and related transcriptional programs, creating an environment that facilitates activation of alternative pro-angiogenic and pro-invasive pathways (17–19). Stress 2: Endogenous signaling stress (direct signal rewiring). A key component of our working model is that anti-VEGF agents can act directly on tumor cells, abrogating pro-survival signaling (an “accelerator” effect) while simultaneously releasing invasion-suppressive constraints (a “brake” effect)—for example, via dissociation of VEGFR2–MET heterocomplexes—thereby promoting reconfiguration of the RTK network toward MET-dominated invasive phenotypes from within the cell (28, 39). Stress 3: Immune–stromal microenvironmental stress (immune and fibrotic/stromal modulation) (22–24, 76–78). VEGF is itself an immunosuppressive mediator, and its blockade can, in principle, enhance anti-tumor immunity (22, 23, 79, 80). Crucially, emerging evidence reveals that various immune subsets (including T cells and dendritic cells) directly express VEGFRs; thus, VEGF functions not only as an angiogen but as a direct immunosuppressive signal, and its blockade fundamentally rewires intracellular signaling within these immune populations (79, 80). However, prolonged therapeutic pressure and treatment-induced cell death may provoke sterile inflammation and recruit myeloid populations (e.g., MDSCs) that possess both immunosuppressive and pro-angiogenic functions (22–24). Concomitantly, anti−VEGF−induced perturbation of vascular and perivascular niches can activate cancer−associated fibroblasts (CAFs) and promote extracellular matrix (ECM) remodeling and stiffening, which further constrains perfusion and facilitates invasive programs (76–78). Cytokines produced by these cells (such as IL-6 and TGF-β) can in turn promote EMT, stemness, and invasion in tumor cells.

Critically, the precise *in vivo* temporal sequence between these stresses—specifically, whether direct signal rewiring (VEGFR2–MET dissociation) acts as an initiating event preceding hypoxia-driven HIF stabilization, or whether they occur concurrently—remains to be empirically defined. Establishing this causality requires dedicated experimental validation. Future studies should employ longitudinal sampling (e.g., serial biopsies) in murine models receiving anti-VEGF therapy, encompassing broad time windows from hours to weeks post-treatment. By combining time-resolved assessment of p-MET, p-VEGFR2, and HIF1-α levels with functional imaging of perfusion and hypoxia, researchers can map the temporal trajectory of “brake release” relative to hypoxic adaptation. Additionally, perturbation experiments manipulating the stability of the VEGFR2–MET complex prior to VEGF blockade would serve as a powerful tool to dissect the hierarchical contribution of direct signaling remodeling within this tripartite framework.

**Figure 2:**
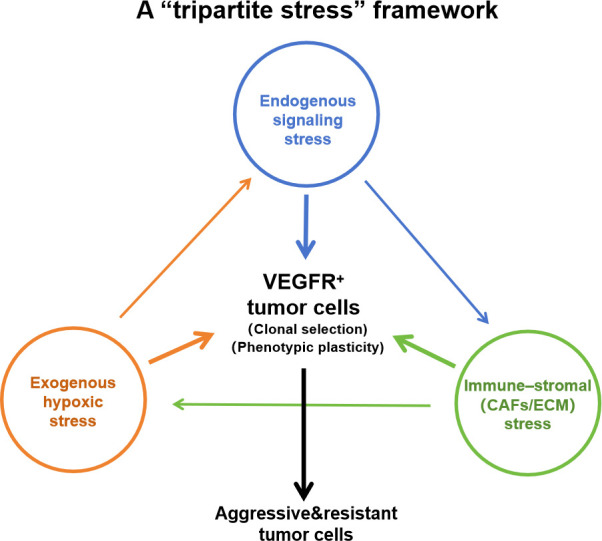
The “tripartite stress” framework for tumor evolution under anti-VEGF therapy. This framework illustrates how three interconnected stresses converge on the VEGFR-expressing (VEGFR^+^) tumor cell population following anti-VEGF treatment. These include: (1) endogenous signaling stress resulting from direct drug effects on tumor cells (i.e., the “brake-accelerator” effect); (2) exogenous hypoxic stress caused by vascular regression; and (3) a composite immune-stromal microenvironmental stress. This latter stress arises from the dynamic remodeling of not only immune cells and cytokines but also the stromal compartment, including key effectors like CAFs and the restructured ECM. The colored arrows connecting the three stress nodes represent a cyclic, mutually reinforcing network of crosstalk: for example, hypoxic stress exacerbates intracellular signaling pressure; altered signaling promotes stromal activation (recruiting CAFs); and a remodeled, stiff ECM further aggravates hypoxia by compressing residual vessels. Collectively, these forces drive clonal selection and phenotypic plasticity, ultimately promoting the emergence of aggressive and resistant tumor cells.

These three stresses are not independent but mutually reinforcing, forming positive-feedback loops. For example, hypoxia upregulates MET and NRP1/2, amplifying the consequences of signal rewiring; rewiring-driven EMT and acquisition of stem-like traits increase secretion of IL-6, CCL2 and promote CAFs activation and ECM remodeling/stiffening, which recruits myeloid cells and strengthens TGF-β/signal transducer and activator of transcription 3 (STAT3) signaling (81, 82); myeloid and TGF-β-driven immune/fibrotic programs activate AXL/FAK/PI3K pathways (83, 84), further promoting invasion and vascular co-option and thereby sustaining hypoxia and bypass angiogenesis. The relative timing and dominance of these stresses are likely context− and tumor−type−dependent, but together they form a multidimensional, positive−feedback cooperation that efficiently selects and sculpts tumor cell populations that escape therapy and display high invasive potential (**Figure 2**).

From this model we derive several falsifiable predictions: (1) concomitant inhibition of MET during VEGF blockade should markedly attenuate the invasion/EMT rebound observed with anti-VEGF monotherapy; (2) increases in hypoxia markers [HIF-1α, carbonic anhydrase IX (CA9)] should coincide with elevated p-MET and upregulation of NRP1/2 and with imaging-detectable infiltrative recurrence; (3) higher MDSC burden and elevated TGF-β/IL-6 levels should predict enhancement of EMT/stemness features and clinical resistance. At an operational level, the model supports using dynamic biomarkers (e.g., phosphorylated MET (p-MET), circulating tumor DNA (ctDNA), evidence of MET amplification, extracellular vesicle (EV)-associated VEGF, NRP1/2 expression, and MDSC/TGF-β/IL-6 levels) as triggerable readouts to stratify patients for addition or sequencing of MET/AXL/NRP or TGF-β pathway inhibitors. These candidates differ in their current levels of clinical validation and should be prioritized and categorized accordingly. It further argues for aligning anti-VEGF dosing with the “vascular normalization window” as suggested by preclinical and imaging studies and for optimizing the temporal integration of anti-angiogenic therapy with immunotherapy.

## 4 Counterexamples, heterogeneity, and boundary conditions

Any biological model has its applicability boundaries. The “direct signal remodeling” model proposed in this paper is no exception; the intensity and manifestation of its effects are constrained by various factors, which are crucial for the precise application of the model.

### 4.1 Cancer-specific signal remodeling and modes of aggressive evolution

In clear cell renal cell carcinoma (ccRCC), upregulation of AXL/MET and an enhanced mesenchymal phenotype are frequently reported after prolonged VEGF axis inhibition (85). Clinically, analysis of tumor tissues from patients with TKI-resistant ccRCC has confirmed that resistance to agents like sunitinib or sorafenib is actively driven by the downregulation of the tumor suppressor MCPIP1 and the concomitant activation of c-Met signaling, which directly promotes metastatic progression (86). The clinical benefit of multi-targeted tyrosine kinase inhibitors (TKIs) like cabozantinib, which directly inhibit MET/AXL, is consistent with this biological directionality (87, 88). This suggests that if the “MET-AXL brake” can be simultaneously applied after “releasing the VEGF brake,” the net effect of aggressive rebound might be reduced. Such observations provide a biological rationale for preventive or trigger-based combinations utilizing dynamic biomarkers (e.g., p-MET, AXL expression, and NRP spatial co-localization).

In CRC, compensatory FGF/FGFR and HGF/MET signaling, along with vessel co-option and infiltrative transformation, are observed after anti-VEGF therapy, particularly in liver metastases (89, 90). This phenomenon suggests that “non-angiogenesis-dependent growth” constitutes a critical boundary condition: even if VEGF axis inhibition is further intensified, it may be difficult to reverse the aggressive migratory advantage if FGFR/MET and associated adhesion-migration programs are not simultaneously inhibited. Correspondingly, FGFR activation markers and histological vessel co-option phenotypes should be incorporated into future stratification and monitoring strategies. Conceptually, this represents a vascular-pattern boundary condition within the tripartite-stress framework, where co-option-driven invasion can partially replace hypoxia-driven bypass angiogenesis.

In breast cancer (BC), particularly in triple-negative breast cancer (TNBC) and other Human Epidermal Growth Factor Receptor 2 (HER2)-negative subtypes, the association of AXL and NRP2 with invasion/metastasis and immunosuppression is especially prominent in several cohorts and preclinical models (91, 92). In a hypoxic-VEGF context, the interaction of Ephrin/AXL/NRP can act as an “amplifier” of the plasticity network, enhancing EMT, stemness, and immune evasion (93, 94). This implies that if VEGF is merely suppressed in a single dimension without constraining the NRP2/AXL hub, an enhanced phenotype due to “pathway diversion” might occur. In combination strategies, attention should be paid to the spatial localization and dynamic levels of NRP2 and AXL, serving as trigger signals for the addition of inhibitors.

In hepatocellular carcinoma (HCC), FGFR4/FGF19 and MET are often found to be activated in parallel after anti-angiogenic therapy, with emerging evidence linking this to reprogramming related to lipid and bile acid metabolism and immune evasion (95–97). This “growth factor-metabolism-immunity” linkage characteristic suggests that continuous inhibition of a single axis can easily be offset by parallel circuits. Therefore, the timed addition of inhibitors targeting FGFR4/FGF19 or MET needs to be accompanied by dynamic co-reading of metabolic and immune biomarkers (e.g., FGF19 levels, tumor-infiltrating CD8^+^ cells, and MDSC burden).

In NSCLC, in EGFR wild-type or kirsten rat sarcoma viral oncogene homolog (KRAS)-mutated populations, the combined benefit of VEGF inhibition and immunotherapy has been associated with baseline or treatment-induced HGF/MET activity (72, 98), which aligns with the “vascular-immune-bypass” tripartite stress model. At baseline, elevated HGF/MET activity may indicate a greater dependence on bypass circuitry and thus a higher rationale for upfront combination strategies. During therapy, treatment-induced rises in p-MET or ctDNA-MET amplification can serve as triggerable thresholds for the addition or sequencing of MET/NRP/AXL inhibitors to achieve feed-forward blockade of aggressive rebound. Strategically, during the early “vascular normalization window,” T cell entry can be optimized.

Counterexamples and boundary cases are also informative. In some tumor types or metastatic niches with low intrinsic dependence on VEGF autocrine signaling, or with pre-existing non-angiogenic growth programs, anti-VEGF therapy may predominantly yield vascular normalization or immune activation without a marked invasive rebound (99). Conversely, in lesions dominated by vessel co-option or alternative vascularization modes, the explanatory power of direct VEGF–RTK rewiring may be reduced unless coupled to stromal and vascular-pattern readouts (89, 100).

In summary, this cross-cancer analysis suggests that after VEGF axis inhibition, the “accelerator effect” of MET/AXL/FGFR and NRP hubs, along with histological vessel co-option, jointly shape aggressive adaptation. The predominant bypass pathways and ecological boundaries differ among cancer types, emphasizing the complexity and application specificity of the model in different clinical contexts (**Table 1**).

**Table 1:** Heterogeneity of resistance to anti-VEGF therapy and precision response strategies.

Cancer type	Dominant evasion mechanisms	Key dynamic biomarkers	Mechanism-driven strategy
ccRCC	Upregulation of MET/AXL signaling; Enhanced mesenchymal phenotype.	Tissue p-MET/p-AXL levels; ctDNA for MET/AXL gene alterations.	Combination with multi-target TKIs inhibiting MET/AXL; Simultaneously blocking the “released brake” and the “activated accelerator”.
CRC	Vessel co-option in liver metastases; Compensatory activation of FGF/FGFR and HGF/MET signaling.	Histological vessel co-option phenotype; FGFR/MET activation markers; Exosomal VEGF.	Combination with MET/FGFR inhibitors in metastatic settings; Strategies to overcome non-angiogenesis-dependent growth.
NSCLC	HGF/MET pathway activation drives resistance and vascular remodeling; Correlates with response to anti-VEGF + ICI.	Tissue p-MET; ctDNA MET amplification; HGF levels; Immune microenvironment status (MDSCs, CD8^+^ T cells).	Combine with ICI during the “vascular normalization window”; Trigger combination or sequencing of MET inhibitors based on biomarker thresholds.
HCC	Parallel activation of FGFR4/FGF19 and MET pathways; Linked with metabolic-immune reprogramming.	Serum FGF19 levels; p-MET; Metabolomic/immunomic biomarkers.	Timed addition of FGFR4 or MET inhibitors; Exploring multi-modal strategies targeting signaling, metabolism, and immunity.
BC	AXL and NRP2 are tightly associated with invasion, metastasis, and immunosuppression.	Expression and spatial co-localization of AXL/NRP2 in tissue.	Co-targeting the NRP2/AXL hub alongside VEGF inhibition.

### 4.2 Model universality and clinical considerations

First, there is universal heterogeneity in the intrinsic tumor context. The expression levels, spatial localization, and dependence on VEGF signaling of VEGFR/MET/NRP vary significantly across different tumor types and even different molecular subtypes within the same tumor, which determines the intensity of the “direct signal remodeling” effect. For example, in GBM and ovarian cancer, the VEGFR2-MET complex-mediated “brake” mechanism has the strongest experimental evidence (28, 39), but its universality and functional intensity in other cancer types still require broader validation, which corroborates the cross-cancer heterogeneity evidence we reviewed in Section 4.1.

Second, differences in drug mechanisms are a key limiting condition for understanding clinical phenomena. When discussing the direct effects of anti-VEGF therapy, it is crucial to precisely distinguish specific drug types, as they play distinct roles in the “brake-accelerator” model. For instance, the humanized anti-VEGFA monoclonal antibody bevacizumab primarily neutralizes extracellular VEGF, leading to the “release of the brake” (101). Given their high structural and functional similarity and comparable clinical efficacy and safety reported for approved bevacizumab biosimilars, these agents are expected to produce largely similar pharmacodynamic effects, consistent with the same “brake-release” and downstream signal-remodeling logic. Small molecule TKIs, on the other hand, present a more complex picture. Some TKIs, such as apatinib, primarily target VEGFR. Because they can penetrate cell membranes, they may suppress intracellular VEGFR activity to some extent; their “brake-release” effect is similar to bevacizumab, but they may exert a stronger inhibitory effect on endogenous survival signals (102). More unique are multi-target TKIs, such as cabozantinib, which are themselves potent inhibitors of both VEGFR and MET (103, 104). The mechanism of action of these drugs is highly specific: while they inhibit VEGFR2, leading to the “release of the brake,” they simultaneously directly inhibit the liberated MET pathway. This is equivalent to an operation within a complex mechanical system that simultaneously releases brake A and applies brake B. This unique dual inhibitory effect means that their clinical manifestations and resistance mechanisms may be distinctly different from drugs that solely target VEGF. Therefore, the specific patterns of “direct signal remodeling” induced by different drugs vary significantly. This clinical concordance supports, rather than proves, our model and highlights the importance of biomarker-guided attribution, as cabozantinib’s benefit is likely multifactorial. It underscores our central thesis: a drug’s clinical behavior is dictated by its precise impact on the tumor cell’s internal signal network. Simply classifying all anti-angiogenic drugs as one category is insufficient; only by delving into the tumor cell level and precisely understanding the direct interaction patterns between specific drugs and intracellular signaling networks (especially key nodes like VEGFR-MET) can we truly predict and ultimately overcome therapeutic resistance.

Finally, the complexity of clinical outcomes also requires comprehensive consideration. Not all patients receiving anti-VEGF therapy will exhibit enhanced aggression. The “vascular normalization window” theory—which posits that at specific doses and times, anti-VEGF therapy can suppress rather than promote invasion by repairing distorted tumor vascular structures, thereby improving oxygen supply and drug delivery (105, 106)—does not contradict our model but rather defines its temporal and dose-dependent boundaries. It suggests that therapeutic pressure is a continuum: below a certain threshold, it may lead to beneficial normalization; above it, it triggers the “tripartite stress” and adaptive resistance cascade we describe. Understanding where a patient’s tumor lies on this continuum is therefore a key clinical challenge. Defining this patient-specific threshold will likely require longitudinal vascular, hypoxia, RTK-rewiring, and immune–stromal readouts (see **Table 2**). Furthermore, differences in the immune–stromal tumor microenvironment (e.g., stromal cell types, initial immune cell infiltration status) also greatly influence the ultimate therapeutic response (99, 107). These factors, alongside the dose- and time-dependent nature of the therapeutic pressure, collectively constitute the complex, context-dependent boundaries for understanding and applying the model presented in this paper.

**Table 2:** Comparison of anti-VEGF agents within the “brake-accelerator” framework.

Drug class (representative)	Monoclonal antibody (Bevacizumab)	Selective VEGFR TKI (Apatinib)	Multi−target TKI (Cabozantinib)
Mechanism of action	Neutralizes extracellular VEGF−A; blocks binding to cell−surface VEGFR1/2.	Cell−permeable TKI inhibiting VEGFR2 kinase activity and downstream signaling.	Cell−permeable multi−kinase TKI inhibiting VEGFR2 and escape kinases, notably MET.
Penetration of intracrine signaling	Low (non−cell−permeable; limited impact on organelle−associated VEGF/VEGFR).	Moderate to high (cell−permeable; can inhibit intracellular VEGFR activity, context−dependent).	High (cell−permeable; targets intracellular VEGFR2 and MET signaling components).
Predicted effect on “Brake” (VEGFR2-MET)	Favors weakening/loss of the brake (mainly indirect)	Favors weakening/loss of the brake; also suppresses VEGFR2 signaling nodes	VEGFR2 suppression can promote brake loss pressure, but MET inhibition limits downstream consequences.
Predicted effect on “Accelerator” (MET/AXL)	No direct inhibition; MET/AXL compensation more likely.	No direct inhibition; MET/AXL remains available as an escape route.	Direct MET inhibition; AXL inhibition depends on target spectrum and context.
Implied resistance mechanisms	Hypoxia adaptation; invasion/EMT; RTK bypass (MET/AXL/FGFR/EGFR); NRP hub signaling; vessel co−option; stromal and immune remodeling.	MET/AXL activation; RTK switching (FGFR/EGFR/PDGFR); hypoxia adaptation; selection/plasticity; incomplete suppression with ligand persistence or parallel pathways.	Non−MET bypass (FGFR/EGFR, etc.); phenotypic switching/plasticity; microenvironmental immunosuppression; exposure limited by toxicity; rewiring to other kinases.

## 5 Clinical implications and future directions

To bridge the gap from our proposed model to improved patient outcomes, this section outlines future strategies. Crucially, we aim to establish translational realism as a cornerstone by addressing not only the strategic rationale for novel biomarkers and combinations but also the practical hurdles inherent in their clinical implementation, including feasibility, standardization, and toxicity management. Based on the “direct signal remodeling” and “tripartite stress” models, we can propose the following specific directions for optimizing anti-VEGF therapeutic strategies.

### 5.1 Next-generation dynamic biomarkers

Future efficacy prediction and resistance monitoring should shift from single, indirect serum VEGF levels to multi-dimensional, dynamic biomarkers that better reflect the intrinsic state of tumor cells. Importantly, these candidates differ in their current levels of clinical validation and should be tiered and prospectively verified in biomarker−integrated trials (**Tables 1**, **2**). The focus of detection should shift to the tumor tissue itself, including the use of immunohistochemistry or spatial transcriptomics to assess the levels of p-MET/total MET, and the expression and spatial co-localization of VEGFR/NRP on tumor cells before treatment. However, recognizing that spatial readouts are currently research-grade and require rigorous standardization before clinical deployment, and that repeated tissue biopsies are often limited by invasiveness and patient compliance, dynamic liquid biopsy emerges as a critical alternative. During treatment, monitoring changes in MET amplification in ctDNA in plasma, or detecting the levels of VEGF protein encapsulated in exosomes that can evade antibody neutralization, as suggested by preclinical and early translational studies (e.g., EV−bound VEGF that is bevacizumab−insensitive) (108). Mechanistically, these extracellular vesicles can be internalized by tumor cells via endocytosis, thereby delivering VEGF to intracellular compartments to sustain intracrine signaling, potentially bypassing surface blockade. This may provide sensitive early signals for warning of aggressive transformation and guiding treatment adjustments.

### 5.2 Precision combination and timed dosing strategies

Managing the balance between efficacy and toxicity is paramount when strictly translating these models into practice. The most direct inference from this model is that key escape pathways should be preventatively blocked concurrently with anti-VEGF therapy. Precision combination therapy based on biomarkers is a core strategy; for example, for patients with high baseline MET expression or MET activation during treatment, MET inhibitors (e.g., Cabozantinib, Crizotinib) may be considered early in combination. However, the combination of anti-VEGF agents with multi-target TKIs creates a risk of overlapping toxicities (e.g., exacerbated hypertension, proteinuria, or fatigue). Additionally, combination strategies targeting other key hubs like NRP1/2 are also worthy of exploration, although the druggability and safety profiles of clinical−grade NRP inhibitors remain to be established. Concurrently, innovative treatment timing and dosing strategies should be explored. Sustained high-intensity VEGF blockade may create the strongest selective pressure. Therefore, low-dose metronomic dosing based on the “vascular normalization” theory, or “adaptive therapy” aimed at controlling rather than eradicating tumors, by intermittent dosing to slow down selective pressure, may maintain efficacy while maximally delaying the emergence of aggressive resistance (105, 106). This rationale is supported by preclinical work indicating that lower, normalization−range anti−angiogenic dosing can sustain vascular function and reduce immunosuppressive rebound relative to prolonged high−dose blockade (99).

Building on this, the combination of anti-VEGF and immune checkpoint inhibitors (ICIs) has also shown clear mechanistic complementarity and significant clinical benefits. The short-term “vascular normalization window” can alleviate tumor hypoxia, reduce the accumulation of MDSCs and regulatory T cells (Tregs), improve dendritic cell maturation and CD8^+^ T cell infiltration, and mitigate immune exclusion caused by endothelial barrier dysfunction and adhesion molecule imbalance, thereby creating a more favorable environment for immune attack (107, 108). The magnitude of this synergy is dose−, timing−, and cancer−type−dependent, consistent with the normalization−to−stress continuum proposed in our model. Clinically, multiple key studies have confirmed the effectiveness of this combination strategy. For instance, the IMpower150 study demonstrated that in NSCLC, atezolizumab+bevacizumab+chemotherapy significantly improved PFS and OS compared to the control group, with patients in the liver metastasis subgroup benefiting particularly, providing strong external validation for “vascular-immune coupling” (109). In RCC, multiple Phase III studies (e.g., KEYNOTE-426 with Axitinib+Pembrolizumab, JAVELIN Renal 101 with Axitinib+Avelumab, CLEAR with Lenvatinib+Pembrolizumab, and CheckMate 9ER with Cabozantinib+Nivolumab) have all demonstrated significant efficacy advantages in first-line treatment (110–113). This evidence collectively supports a strategy where, when the VEGF axis is inhibited, synchronous immune activation is employed to counteract the combined pressure of bypass activation and immunosuppression. Based on the “Tripartite Stress” framework, we suggest considering lower doses or metronomic anti-VEGF therapy during the early vascular normalization window to optimize T cell infiltration. Simultaneously, based on dynamic biomarker thresholds (e.g., rising p-MET, complex dissociation, ctDNA-MET amplification), MET/NRP/AXL inhibitors can be layered or adjusted in sequence. Crucially, this dynamic regulation of dosing intensity and sequencing aims to achieve a “feed-forward blockade” of aggressive rebound while explicitly mitigating the cumulative toxicity observed in concurrent distinct-pathway inhibition.

### 5.3 Key considerations for future clinical trial design

To translate the above theories into clinical benefits, future clinical trial designs should be adjusted accordingly. It is recommended that early Phase I/II studies prospectively integrate dynamic biomarker monitoring. For example, trial protocols should specify collection of blood samples and (where possible) tumor tissue at multiple time points to systematically monitor changes in p-MET, EV-VEGF, and circulating tumor cell (CTC) EMT markers, and correlate them with imaging assessments and clinical outcomes. Furthermore, beyond biomarker monitoring for clinical decision-making, future trials should particularly emphasize the systematic, longitudinal collection of tumor tissue samples from patients after recurrence/progression. Only by performing multi-dimensional comparative analysis, such as single-cell sequencing and spatial transcriptomics (114, 115), on paired pre- and post-treatment samples, can we ultimately dissect the relative contributions of “clonal selection” and “phenotypic plasticity” at the clinical level, thereby providing the highest level of direct evidence for the model proposed in this paper. Additionally, adaptive trial designs based on biomarkers can be attempted (116, 117). While such designs are still largely prospective proposals in anti−VEGF settings, they provide a practical route to test the model’s trigger−based combination logic. For example, design a trial where all patients initially receive a standard anti-VEGF regimen. When a biomarker (e.g., p-MET) exceeds a preset threshold, patients are randomized into either the group continuing the original regimen or the group combining with a MET inhibitor. This design can more efficiently validate the value of combination strategies and provide high-level evidence for individualized treatment pathways.

## 6 Conclusion

Aggressive resistance emerging after anti-VEGF therapy is a central obstacle limiting its clinical application potential. The “direct signal remodeling” model, and its extended “tripartite stress” framework, presented in this paper, emphasize that academia and clinicians need to focus more on tumor cells themselves, beyond the traditional “angiocentric” view. As a working prototype rather than a universal mechanism, this framework encourages us to recognize that anti-VEGF drugs may not merely “starve” tumors but also actively and directly remodel the intrinsic signaling networks of tumor cells, resulting in complex dual effects of “accelerator” inhibition and “brake” release. This process, under the synergy of hypoxic and immune–stromal microenvironmental pressures, driven by clonal selection and phenotypic plasticity, can lead to the malignant evolution of tumors, with the relative timing and dominance of each stress likely varying across tumor types and treatment contexts. Future research should focus on utilizing multi-omics, spatially resolved, and dynamic monitoring biomarkers to precisely delineate this process, and accordingly develop mechanism-based precision combination therapies and innovative adaptive treatment strategies, ideally validated through longitudinal paired-sample studies and trigger−based adaptive trials. Only then can we truly wield this “double-edged sword” of anti-VEGF therapy, through biomarker−guided, dose− and timing−adapted, cancer−type−specific approaches, maximizing its therapeutic potential to bring more lasting and profound survival benefits to a wide range of cancer patients.

## Data Availability

The original contributions presented in the study are included in the article/supplementary material. Further inquiries can be directed to the corresponding author.
